# Solid state lithiation–delithiation of sulphur in sub-nano confinement: a new concept for designing lithium–sulphur batteries†
†Electronic supplementary information (ESI) available. See DOI: 10.1039/c5sc03419a


**DOI:** 10.1039/c5sc03419a

**Published:** 2015-11-10

**Authors:** Chengyin Fu, Bryan M. Wong, Krassimir N. Bozhilov, Juchen Guo

**Affiliations:** a Department of Chemical and Environmental Engineering , University of California Riverside , Riverside , CA 92521 , USA . Email: jguo@engr.ucr.edu; b Materials Science and Engineering Program , University of California Riverside , Riverside , CA 92521 , USA; c Central Facility for Advanced Microscopy and Microanalysis , University of California , Riverside , CA 92521 , USA

## Abstract

We investigate the detailed effects and mechanisms of sub-nano confinement on lithium–sulfur (Li–S) electrochemical reactions in both ether-based and carbonate-based electrolytes. Our results demonstrate a clear correlation between the size of sulfur confinement and the resulting Li–S electrochemical mechanisms. In particular, when sulfur is confined within sub-nano pores, we observe identical lithium–sulfur electrochemical behavior, which is distinctly different from conventional Li–S reactions, in both ether and carbonate electrolytes. Taken together, our results highlight the critical importance of sub-nano confinement effects on controlling solid-state reactions in Li–S electrochemical systems.

## Abstract 2

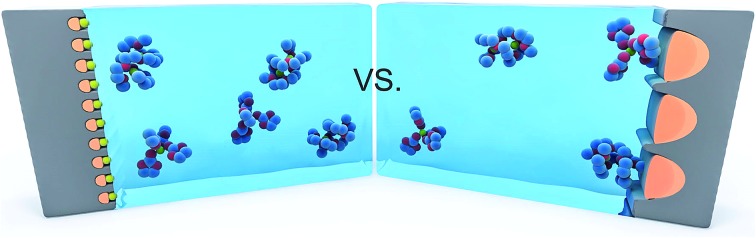
Sub-nano confinement of sulphur enables a solid-state lithium–sulfur electrochemical reaction mechanism in liquid electrolytes.

## Introduction

Rechargeable lithium–sulphur (Li–S) batteries continue to be one of the most promising technologies for electrochemical energy storage. In order to dramatically improve the performance of these Li–S systems, we require a detailed understanding of the interactions between lithium and sulphur in these complex, heterogeneous electrochemical environments. Due to the high electrical resistivity of sulfur,^1^ it is essential to incorporate sulphur into conductive hosts, of which the majority are carbonaceous materials. The rationale for using carbonaceous hosts, particularly porous carbon materials, is rooted from the well-known polysulfide shuttle reaction induced by the dissolution of lithium polysulfides (Li_2_S_*n*_) into electrolytes. To date, it is widely accepted that only ether-based electrolytes are feasible for Li–S batteries. The two most common ones are tetra(ethylene glycol) dimethyl ether (TEGDME) and a mixture of 1,3-dioxolane (DOL) and 1,2-dimethoxyethane (DME). These solvents can efficiently solubilize lithium polysulfides, which is necessary to achieve an in-depth lithiation of sulphur. However, at the same time the polysulfide dissolution also causes problematic characteristics, so-called “shuttle reactions.” Our measurements show that the solubility of Li_2_S_8_ in TEGDME at room temperature is very high as 0.18 ± 0.005 M (equivalent to 1.44 M of sulphur); *i.e.* approximately 22 mL of TEGDME electrolyte can completely dissolve Li_2_S_8_ generated from 1 g of S_8_. As a result, most of the Li–S batteries are essentially batteries with “liquid phase” cathodes – upon lithiation, the initial product (Li_2_S_8_) immediately dissolves with high local concentration at the cathode–electrolyte interface. Products from further lithiation have distinctly lower solubility so that precipitation/deposition of Li_2_S_*n*_ (*n* < 8) on the cathode sequentially occurs. Therefore, the complex Li–S electrochemical processes at the cathode involve generation, disappearance, and migration of multiple electroactive species both in the solution and on the electrode. As clear evidence, lithium polysulfide generation and re-distribution during the first discharge was recently observed *via in situ* techniques including Raman spectroscopy,^2^ transmission X-ray microscopy,^3^ and X-ray fluorescence microscopy.^4^

Certainly, the complex Li–S electrochemical processes in these systems can be further tailored to achieve enhanced battery performance. One effective strategy, pioneered by Nazar and coworkers,^5^ is to employ porous structures as sulphur hosts and polysulfides reservoirs. Many porous cathode structures including amorphous porous carbons,^6^–^9^ core–shell structures,^10^–^13^ carbon nanotube networks,^14^–^16^ and porous structures composed of graphene/graphene oxide^17^–^22^ have been investigated. Another viable strategy is the “catholyte” concept.^23^–^27^ Instead of sequestering lithium polysulfides in the cathode, catholyte Li–S cells use electrolytes with a high concentration of dissolved lithium polysulfides, and excellent battery performance was achieved by optimizing the concentration and composite of the catholytes. The third strategy is to chemically modify the cathode hosts to render strong adsorption to the lithium polysulfides species. Heteroatoms in the carbon matrix, including nitrogen and oxygen, have been proven effective.^28^–^31^

In spite of these impressive improvements, a fundamental question of both scientific and technological importance remains: is it possible to restrict the electroactive sulphur-containing species in the solid state during the Li–S electrochemical reaction? If possible, this hypothesized solid-state Li–S electrochemical reaction would have transformative implications for altering the electrochemical processes and performance of Li–S batteries.

## Results and discussion

**Fig. 1:**
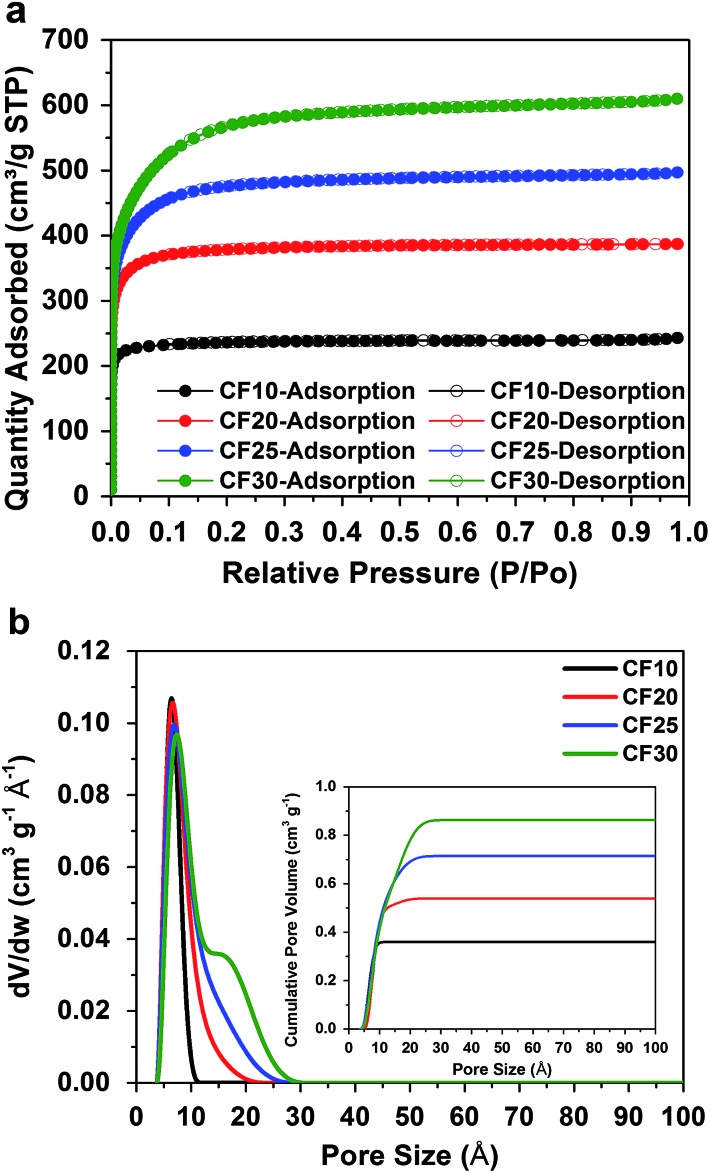
(a) Type-I nitrogen adsorption–desorption isotherms and (b) pore size distributions (cumulative pore volume as inset) of the four different carbon fibres.

To answer this question, we investigate two factors that play decisive roles in Li–S electrochemical processes: the size of the sulphur confinement (*i.e.* pore size in the carbon hosts) and the type of electrolyte solvents. To precisely capture the subtle changes in Li–S electrochemical behaviour due to the different sulphur confinement size, a series of porous carbon hosts with narrow ranges of pore sizes is selected: resorcinol-formaldehyde derived porous carbon fibres with four distinctly different pore sizes, 0.4–1.0 nm, 0.4–2.0 nm, 0.4–2.5 nm, and 0.4–3.0 nm (denoted as CF10, CF20, CF25, and CF30), respectively, were purchased from Kuraray Chemical Co., Ltd. The scanning electron microscope (SEM) images of these carbon fibres are shown in Fig. S1 in ESI.† Fig. 1a shows the type-I nitrogen adsorption–desorption isotherms of these four carbon fibbers, and Fig. 1b shows their pore size distributions calculated based on a non-local density functional theory (NLDFT) model. The detailed structural properties are listed in Table S1 in ESI.†

In addition to the different pore sizes, two different electrolyte solvent systems are selected for this study: the first one is TEGDME, which is a typical solvent for conventional Li–S batteries as aforementioned. The other solvent is a mixture of ethylene carbonate and diethyl carbonate (EC/DEC) with a 1 : 1 volume ratio, which is a typical solvent for Li-ion batteries and is well-known for their failure in conventional Li–S batteries. Commercial grade EC/DEC electrolyte with 1 M lithium hexafluorophosphate (LiPF_6_) salt was purchased from Sigma-Aldrich. Since LiPF_6_ does not dissociate well in ethereal solvents, lithium bis(trifluoromethane sulfonyl)imide (LiTFSI), which has a higher dissociation constant,^32^ was used in TEGDME electrolyte with a concentration of 1 M. The previous study by Abruña and co-workers suggests that the type of lithium salt anion (LiPF_6_*vs.* LiTFSI) does not affect lithium–sulphur electrochemical reactions.^33^ Therefore, the major difference between these two electrolytes is their solubility of lithium polysulfides, particularly for the high order structures. As shown in Table S2 in ESI,† TEGDME is a superior solvent for lithium polysulfides comparing to EC/DEC.

To demonstrate the effects of sulphur confinement on Li–S electrochemical reactions, we first investigated three CF10 (the smallest pore size among all four carbon fibbers) samples with different sulphur contents. Among these samples, CF10–S_60_ and CF10–S_90_ have 60 wt% and 90 wt% of sulphur, respectively, by infusing the designated amount of sulphur through heating the mixture of CF10 and sulphur at 155 °C in argon for 10 hours.

**Fig. 2:**
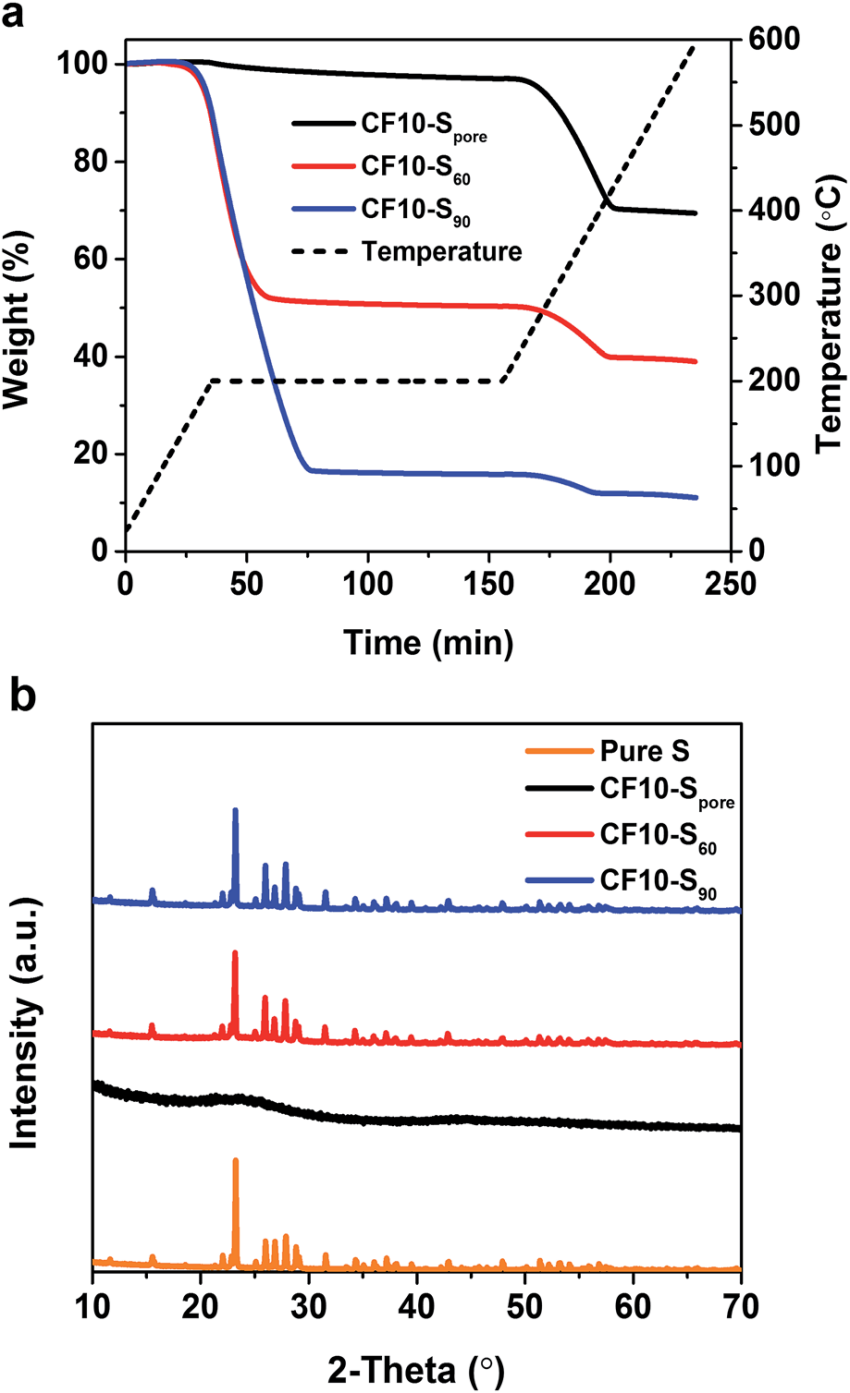
(a) TGA curves and (b) XRD patterns of CF10–S_pore_, CF10–S_60_ and CF10–S_90_.

CF10–S_pore_ was obtained by further heating CF10–S_60_ at 200 °C in flowing argon for 6 hours to remove the sulphur deposited on the surface. Thermogravimetric analysis (TGA) was performed in argon from room temperature to 600 °C with a heating ramp of 5 °C min^–1^, and a 2 hour isothermal step was imposed at 200 °C. As shown in Fig. 2a, the TGA plots clearly show that both CF10–S_60_ and CF10–S_90_ have two weight loss stages: the first weight loss starts with the isothermal step at 200 °C and completes prior to the end of the isothermal step. The second weight loss stage starts at 270 °C and completes at 400 °C. The first weight loss was due to the sublimation of the sulphur deposited on the surface of CF10 (denoted as superficial sulphur), and the second weight loss arises from the sublimation of the sulphur confined in the sub-nano pores of CF10 (denoted as confined sulphur).^6^ In stark contrast, CF10–S_pore_ only demonstrates the second weight loss stage, indicating that the sulphur in CF10–S_pore_ is exclusively confined in the sub-nano pores, and the confined sulphur content in CF10–S_pore_ is 30 wt%, which is consistent with the percentage of confined sulphur in both CF10–S_60_ and CF10–S_90_. The X-ray diffraction (XRD) pattern of CF10–S_pore_ in Fig. 2b indicates that the sulphur confined in the sub-nano pores is amorphous, and the superficial sulphur in CF10–S_60_ and CF10–S_90_ has an orthorhombic crystal structure.

**Fig. 3:**
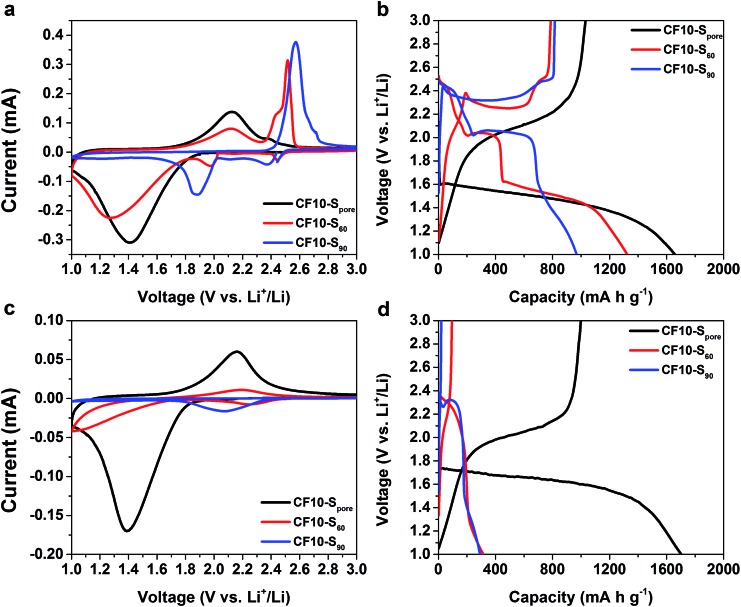
The first CV scans at 0.1 mV s^–1^ and the first GCD curves at 160 mA g^–1^ of CF10–S_pore_, CF10–S_60_, and CF10–S_90_ in TEGDME electrolyte (a) and (b) respectively, and in EC/DEC electrolyte (c) and (d), respectively.

As demonstrated in Fig. 3a and b, the drastically different cyclic voltammetry (CV) and galvanostatic charge–discharge (GCD) characteristics of CF10–S_90_, CF10–S_60_, and CF10–S_pore_ show a clear correlation to the sulphur distribution (superficial *vs.* confined) in the TEGDME electrolyte. The CV scan of CF10–S_90_ demonstrates typical liquid-phase Li–S electrochemical behaviour with two cathodic peaks at 2.40 V and 1.95 V and one anodic peak at 2.6 V (with a shoulder at 2.7 V), which is consistent with the GCD curve of CF10–S_90_ with a lithiation capacity of 750 mA h g^–1^. When the content of superficial sulphur is reduced in CF10–S_60_, its CV scan is rather interesting: in addition to the two aforementioned conventional cathodic peaks representing the liquid-phase lithiation of sulphur, it also shows a broad cathodic peak below 1.8 V. Accordingly, the anodic scan demonstrates a broad peak at 2.1 V in addition to the typical anodic peaks at 2.5 V. The GCD curve of CF10–S_60_ is consistent: it shows the typical sulphur discharge plateaus at 2.45 V and 2.0 V and a pronounced new discharge slope at 1.5 V with a total lithiation capacity of 900 mA h g^–1^. The transition of CV and GCD behaviours becomes more clear when the sulphur content is further reduced in CF10–S_pore_: with sulphur exclusively confined in the sub-nano pores, the conventional Li–S CV peaks completely disappear. Instead, the CV scan of CF10–S_pore_ only shows a single pair of redox peaks centred at 1.4 V and 2.1 V. Accordingly, the GCD curve of CF10–S_pore_ shows a single lithiation slope starting from 1.6 V and a single delithiation slope starting from 1.8 V with a lithiation capacity of 1650 mA h g^–1^.

As shown in Fig. 3c and d, the electrochemical characteristics of CF10–S_90_, CF10–S_60_, and CF10–S_pore_ in EC/DEC electrolyte also show a strong correlation to the sulphur distribution in CF10. The CV scan of CF10–S_90_ in EC/DEC only shows a small cathodic peak centred at 2.1 V and no reversible anodic peak is shown. This observation indicates that the lithiation process of CF10–S_90_ in EC/DEC electrolyte is not only terminated at a very early stage, but is also irreversible. Consistently, the GCD curve of CF10–S_90_ in EC/DEC shows only a very short discharge plateau at 2.3 V and no charge capacity at all, which is consistent with the known behaviour of Li–S electrochemical reaction in carbonate electrolytes.^33^,^34^ The first cathodic scan of CF10–S_60_ in EC/DEC also shows a small peak at 2.2 V with an additional small peak below 1.8 V, which corresponds to a broad anodic peak at 2.1 V. The GCD curve of CF10–S_60_ in EC/DEC consistently shows a small discharge slope from 2.3 V with a modest capacity of 300 mA h g^–1^ that is partially reversible. This observation suggests that CF10–S_60_ still has a very low electrochemical activity in EC/DEC electrolyte, although improved from CF10–S_90_. When the superficial sulphur is completely removed, however, the electrochemical behaviour of CF10–S_pore_ is strikingly different: its CV scan in EC/DEC shows a single pair of redox peaks at 1.4 V and 2.1 V, which is identical to the CV scan of CF10–S_pore_ in the TEGDME electrolyte. The GCD curve of CF10–S_pore_ in EC/DEC electrolyte shows a single lithiation slope starting from 1.7 V and a single delithiation slope starting from 1.8 V with a lithiation capacity of 1700 mA h g^–1^, which is also nearly identical with that in the TEGDME electrolyte. The sequential CV scans and GCD cycles of all three samples in both electrolytes are all consistent as shown in Fig. S2 in ESI.†

It is clear that the electrochemical behaviour of these three samples are determined by the surrounding environment of the sulphur, *i.e.* confined sulphur *vs.* superficial sulphur. When superficial sulphur is present, only TEGDME (an ether) is a viable electrolyte solvent for enabling conventional liquid-phase Li–S electrochemical processes. However, when the sulphur is exclusively confined in the sub-nano pores, both TEGDME and EC/DEC (carbonates) can facilitate identical Li–S electrochemical reactions. These anomalous electrochemical behaviours of sulphur were scarcely investigated in some previous studies using microporous carbons as sulphur hosts, and a few mechanisms have been proposed.^34^–^41^ One generally accepted hypothesis ascribes these anomalous behaviours to the lithiation and delithiation of small sulphur allotropes such as S_4_ or S_2_ in the sub-nano pores.^36^–^40^ Another hypothesis attributes these anomalous behaviours to the lithiation and delithiation of sulfurized carbon.^41^ With the assumption of the existence of small sulphur allotropes (S_2–4_) in the sub-nano pores, Li and co-workers proposed a solid-state Li–S reaction mechanism induced by the prevention of solvent penetration due to the pore size limitation.^40^

We agree with Li and co-workers on the mechanism of solid-state Li–S reactions in sub-nano confinement, although the form of sulphur in sub-nano confinement calls for further studies (ESI†). When sulphur is exclusively confined in very small pores, the electrochemical lithiation and delithiation of sulphur can only occur when Li ions enter the pores. Previous studies of porous carbon capacitors demonstrated that sub-nano pores in carbon might not be accessible to cations in non-aqueous electrolytes due to the larger solvation shell.^42^,^43^ For instance, the size of the Li ion solvation shell in propylene carbonate was estimated as 1.59 nm.^44^ As a result, Li ions can only enter the pores by either desolvation or solvation shell distortion, and there may be none or very few solvent molecules inside the sub-nano pores where the Li–S electrochemical reaction occurs. Meanwhile, the extremely small sulphur grains in sub-nano confinement and the intimate contact with carbon can ensure the in-depth lithiation in solid state. In an analogous experiment, Gogotsi and co-workers discovered an anomalously high capacitance in supercapacitor electrodes made of microporous carbon, and they hypothesized that it was due to ions entering the sub-nano pores *via* desolvation.^45^–^47^ We posit that a similar phenomenon occurs during the lithiation–delithiation of sulphur in very small confinement within liquid electrolytes. This hypothesized mechanism explains why both TEGDME and EC/DEC are viable electrolyte solvents for CF10–S_pore_ with identical characteristics: the solid-state Li–S electrochemical reaction no longer involves the dissolution of polysulfides or prohibited by severe polysulfide/electrolyte incompatibility.^33^ It is worth noting that recent studies on all-solid-state Li–S batteries with ceramic electrolytes^48^,^49^ demonstrated very similar electrochemical behaviours of sub-nano confined sulphur in liquid electrolyte, which also supports our hypothesized solid-state reaction mechanism.

The cycle stability of CF10–S_pore_, CF10–S_60_, and CF10–S_90_ in TEGDME and EC/DEC electrolytes are shown in Fig. S3 in ESI.† The cycle stability of CF10–S_pore_ indicates a slight advantage of EC/DEC electrolyte over TEGDME electrolyte in terms of long–term cycle stability, which was also reported in a previous study.^37^ This observation can be attributed to the fact that the microporous structure of CF10 is not ideal; therefore, lithium polysulfides could be gradually generated and dissolved in the TEGDME electrolyte.

**Fig. 4:**
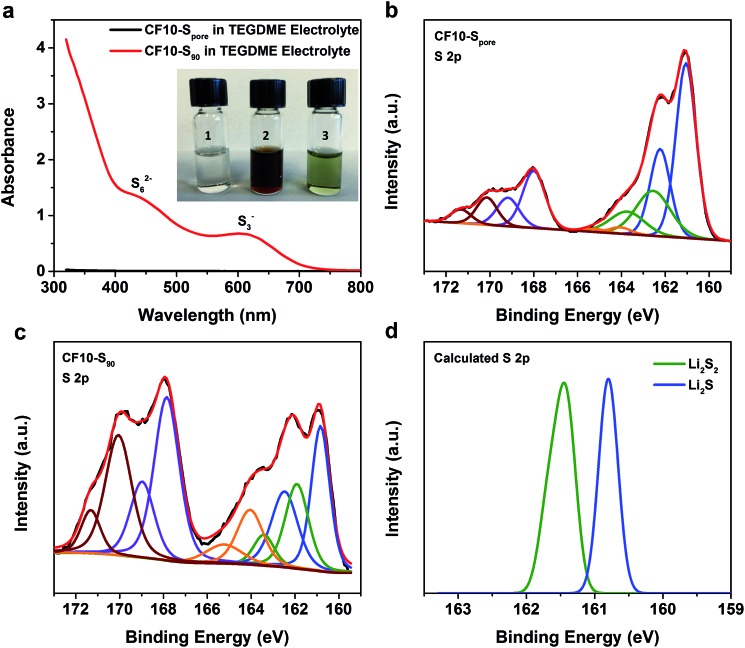
(a) UV-Vis spectra of the TEGDME electrolyte used in CF10–S_pore_ lithiation and the diluted TEGDME electrolyte used in CF10–S_90_ lithiation, inset is photograph of the TEGDME electrolytes used in CF10–S_pore_ and CF10–S_90_ lithiation; XPS spectra of S 2p in (b) lithiated CF10–S_pore_ and (c) lithiated CF10–S_90_; (d) predicted binding energies of sulfur in Li_2_S_2_ and Li_2_S from DFT calculation.

For further evidence of the solid-state lithiation–delithiation mechanism of sub-nano confined sulphur, we performed the following experiments: CF10–S_pore_ and CF10–S_90_ electrodes containing an equal mass of sulphur (∼10 mg) were lithiated in 5 mL TEGDME electrolyte, respectively, in two home-made PTFE cylindrical cells with 80 mA g^–1^ current density to ensure in-depth lithiation. After the lithiation, the TEGDME electrolytes in these two cells were immediately extracted for UV-Vis spectroscopy analysis. The inset in Fig. 4a shows the photographs of the TEGDME electrolytes used in the CF10–S_pore_ lithiation (vial #1) and CF10–S_90_ lithiation (vial #2). It can be clearly seen that the electrolyte used for CF10–S_pore_ has no visible colour change; however, the electrolyte used for CF10–S_90_ becomes dark red, which indicates the presence of lithium polysulfides. To facilitate UV-Vis spectra measurements, the TEGDME electrolyte for CF10–S_90_ was diluted 5 times, and the colour changed from burgundy to ultramarine green (vial #3), which indicates the presence of S_3_^–^ free radical.^50^Fig. 4a shows the UV-Vis spectra of the TEGDME electrolyte for CF10–S_pore_ and the diluted TEGDME electrolyte for CF10–S_90_; lithium polysulfide species including S_6_^2–^ anion and S_3_^–^ free radical were detected in the diluted TEGDME electrolyte for CF10–S_90_.^51^ In contrast, no polysulfide species were detected in the TEGDME electrolyte for CF10–S_pore_. This observation clear demonstrates that no lithium polysulfides are dissolved into the electrolyte when sulphur is confined in sub-nano pores and, therefore, the lithiation occurs in the solid state.

To analyse the composition of the final products from the lithiation of CF10–S_pore_ and CF10–S_90_ in the TEGDME electrolyte, we further performed XPS analyses on these lithiated electrodes, of which the S 2p spectra are shown in Fig. 4b and c, respectively. Sulphur at any valence state always has split peaks in XPS separated by 1.18 eV arising from S 2p_3/2_ (higher binding energy) and S 2p_1/2_ (lower binding energy) spin–orbit splitting. In our analysis, we index the sulphur species by the higher binding energy of the S 2p_3/2_ peak. The two split-peaks at 167.6 eV (purple) and 170.2 eV (crimson) in both CF10–S_pore_ and CF10–S_90_ can be attributed to the sulphonyl residue from the LiTFSI salt and the sulphur oxide species from sulphur infusion and electrolyte decomposition.^52^–^54^ The three split-peaks in the range from 167 eV to 159.5 eV represent three sulphur species resulting from the lithiation. The first observation is that both CF10–S_pore_ and CF10–S_90_ have same lithiated species at 164.0 eV (orange), ∼162.0 eV (green), and ∼161.0 eV (blue), which can be respectively assigned as un-lithiated sulphur, lithium persulphide (Li_2_S_2_), and lithium sulphide (Li_2_S).^55^ It is not surprising that no other lithium polysulfide species was observed in the lithiated CF10–S_pore_ according to our proposed solid-state lithiation mechanism that does not invlove polysulfides. It is also not surprising to see the same products in the lithiated CF10–S_90_: previous investigations on the Li–S phase diagram clearly demonstrated that elemental sulphur and Li_2_S are the only stable phases in solid state at room temperature.^56^,^57^ Lithium polysulfides spontaneously disproportionate to sulphur and Li_2_S upon drying. Meanwhile, both experimental and theoretical studies indicate Li_2_S_2_ is a metastable phase, which could exist in the solid state at room temperature.^58^–^60^ Despite these similarities, the percentage of each sulphur species in the lithiated CF10–S_pore_ and lithiated CF10–S_90_ are distinctly different as indicated by the peak area (Table S3 in ESI†). Only 2.6% of the lithiated sulphur in CF10–S_pore_ remains as elemental sulphur, and the content of S^2–^ and S_2_^2–^ anions are 67.8% and 29.6%, respectively. On the contrary, 22.8% of the lithiated sulphur in CF10–S_90_ still remains as elemental sulphur, and the content of S^2–^ and S_2_^2–^ anions are 49.5% and 27.7%, respectively. The much lower sulphur content in the lithiated CF10–S_pore_ clearly indicates superior sulphur utilization in the solid-state lithiation mechanism, which is also consistent with the demonstrated higher sulphur-based capacity of CF10–S_pore_ in Fig. 3b.

Based on these results, we find that the size of the sulphur confinement has a profound effect on Li–S electrochemical processes; *i.e.* there is a critical size regime in order for the proposed solid-state Li–S electrochemical reaction to occur. To investigate these effects, another three carbon fibers with different ranges of pore size (CF20, CF25, and CF30) were investigated as sulfur hosts. Samples with sulfur exclusively confined in the pores, namely CF20–S_pore_, CF25–S_pore_, and CF30–S_pore_, were prepared using the same method for CF10–S_pore_ preparation (TGA in Fig. S4 in ESI†). The transmission electron microscopy (TEM) images and the elemental mapping of the cross sections CF10–S_pore_, CF20–S_pore_, CF25–S_pore_, and CF30–S_pore_ (Fig. S5 in ESI†) clearly show that sulfur is uniformly dispersed in all of the carbon fiber samples. The XRD patterns (Fig. S6 in ESI†) indicate that the sulfur in all four confinements is amorphous.

**Fig. 5:**
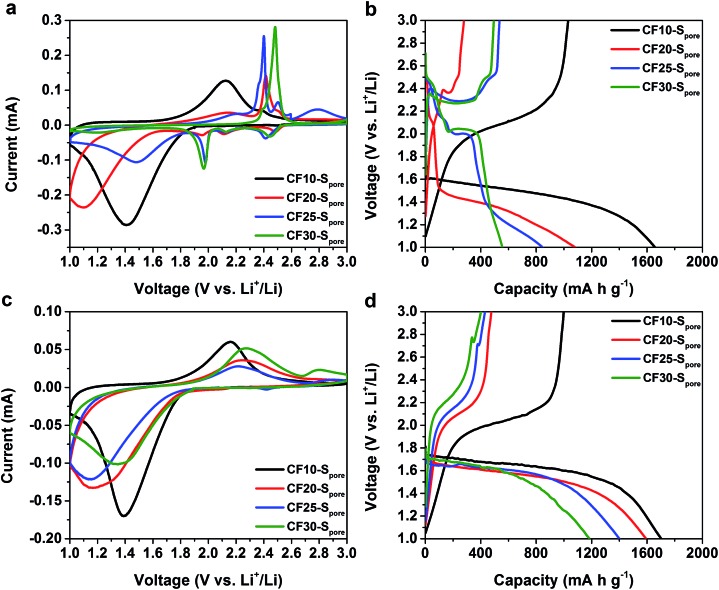
The first CV scans at 0.1 mV s^–1^ and GCD curves at 160 mA g^–1^ of CF10–S_pore_, CF20–S_pore_, CF25–S_pore_, and CF30–S_pore_ in TEGDME electrolyte (a) and (b) respectively, and in EC/DEC electrolyte (c) and (d), respectively.

As we anticipated, the electrochemical characteristics of sulphur indeed show a clear correlation to the confinement size. Fig. 5a and b, respectively, show the first CV scans and the first GCD cycles of CF10–S_pore_, CF20–S_pore_, CF25–S_pore_, and CF30–S_pore_ in the TEGDME electrolyte. As aforementioned, the CV of CF10–S_pore_ shows a single pair of redox peaks. The CV of CF20–S_pore_ shows both the redox peaks representing the liquid phase Li–S electrochemical reactions (cathodic peaks at 2.5 V, 2.2 V, 2.0 V and anodic peaks at 2.4 V and 2.6 V) and the low-potential redox peaks representing the solid-state Li–S reaction. In the CV of CF25–S_pore_, the peak currents of the low-potential redox pair are further reduced, whereas the peaks representing liquid phase Li–S reactions become more dominating. For CF30–S_pore_, its CV demonstrates the characteristics of conventional liquid phase Li–S electrochemical reactions without the low-potential redox peaks. All the CV scans are consistent with their corresponding GCD curves shown in Fig. 5b. Based on the evolution of the characteristics of Li–S electrochemical reactions as a function of the pore size, we posit that the critical size of sulphur confinement for solid-state Li–S electrochemical reaction is about 1.0 nm, *i.e.* sub-nanometre confinement. Fig. 5c and d are the first CV scans and the first GCD cycles of CF10–S_pore_, CF20–S_pore_, CF25–S_pore_, and CF30–S_pore_ in the EC/DEC electrolyte. All of the CV scans and the corresponding GCD curves demonstrate consistent characteristics with a single pair of redox peaks and single lithiation–delithiation slopes. However, compared to CF10–S_pore_, the CV redox pairs of CF20–S_pore_, CF25–S_pore_, and CF30–S_pore_, which have larger pore sizes, demonstrate higher peak separation indicating inferior charge transfer kinetics. Also as shown in Fig. 5d, the lithiation and delithiation capacity drastically decreases with increasing pore size. The decreased capacity can be attributed to the fact that sub-nano confined sulphur population decreases as the pore size range increases from 0.4–1.0 nm to 0.4–3.0 nm. The second CV scans and GCD cycles show consistent characteristics (Fig. S7 in ESI†). The cycle stability results in both EC/DEC electrolyte and TEGDME are shown in Fig. S8 in ESI.†

**Fig. 6:**
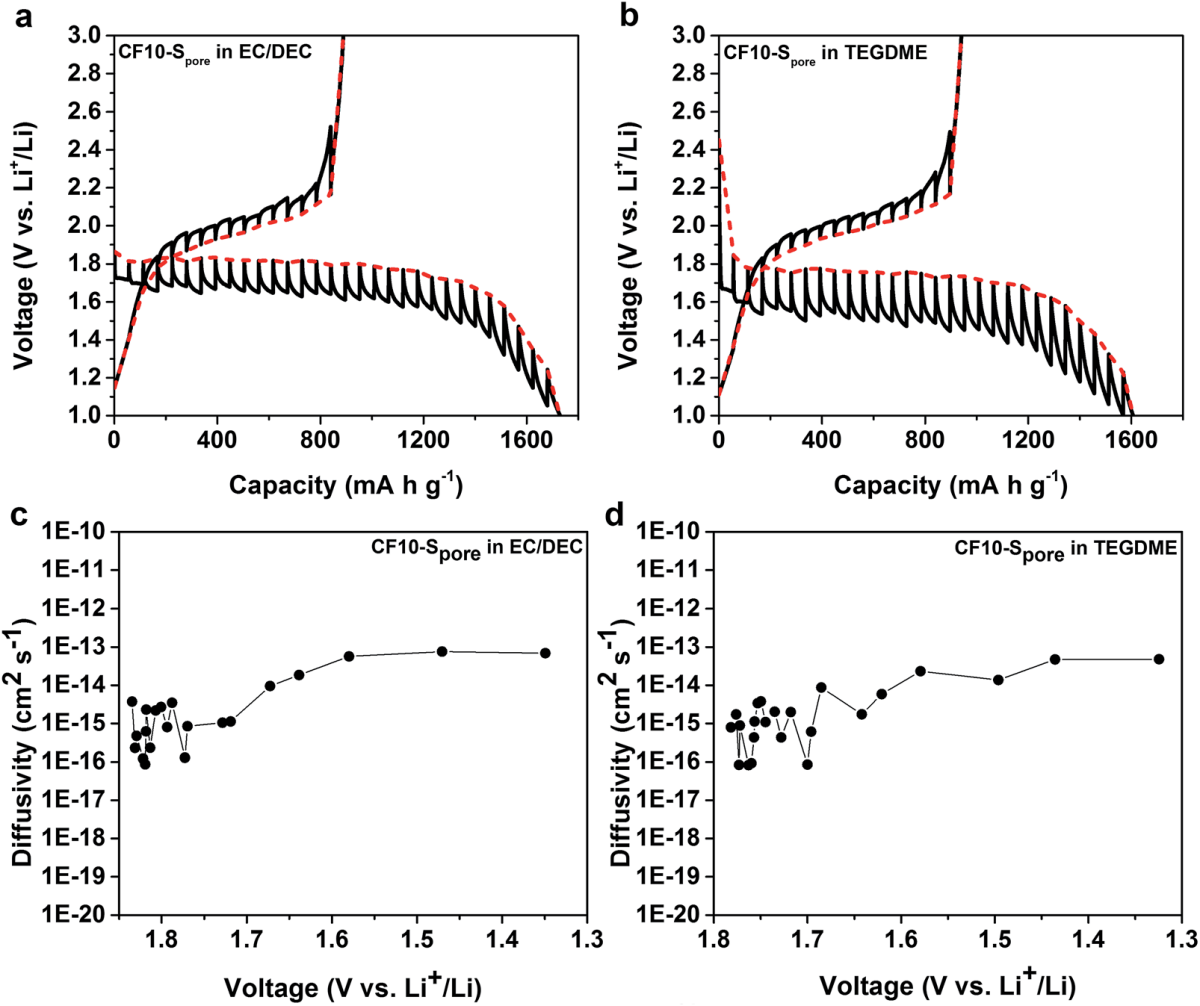
GITT curves of CF10–S_pore_ in (a) EC/DEC electrolyte and (b) TEGDME electrolyte; diffusivity of Li *vs.* potential calculated from the GITT data in (c) EC/DEC electrolyte and (d) TEGDME electrolyte.

We further studied the equilibrium potential of the solid-state Li–S reaction under sub-nano confinement (CF10–S_pore_) using galvanostatic intermittent titration techniques (GITT) as shown in Fig. 6a and b. The GITT results of CF20–S_pore_ and CF25–S_pore_ in both TEGDME and EC/DEC electrolytes are shown in Fig. S9 in ESI.† Comparing the GITT results of CF10–S_pore_ in the EC/DEC electrolyte with that in the TEGDME electrolyte, it is clear that the single lithiation plateau is an inherently thermodynamic characteristic of the solid-state Li–S electrochemical reaction in the sub-nano confinement regardless electrolyte. The equilibrium solid-state lithiation potential is 1.8 V *vs.* Li/Li^+^ in both electrolytes. However, the lithiation overpotential in TEGDME is 280 mV, which is much higher than that in EC/DEC (150 mV). Since the electrodes are identical, the different overpotentials must be due to the electrolytes. One possibility is the different solvation size or solvation energy of Li ions in TEGDME *vs.* EC/DEC: a previous study by Henderson and co-workers suggested the Li-ion solvation structure in TEGDME consisting of two six-coordinate Li cations coordinated by two TEGDME molecules.^61^ Such a double-helix dimer structure can impose energy barriers to the desolvation of TEGDME resulting in a higher overpotential. The solid-state Li diffusivity in sulphur is calculated from the GITT data as shown in Fig. 6c and d as a function of lithiation potential. The average diffusivity is calculated as 1.16 × 10^–15^ cm^2^ s^–1^ as measured in EC/DEC and 1.26 × 10^–15^ cm^2^ s^–1^ as measured in TEGDME in the lithiation slope region, which are in excellent agreement with each other. The Li diffusivity increases when the voltage is decreased below 1.7 V, which can be attributed to the enrichment of Li in the lithiated sulphur. The apparent diffusivity of Li in the liquid-phase Li–S reaction was also estimated from the high-voltage plateau region from CF25–S_pore_ in TEGDME (Fig. S10d in ESI†). The apparent diffusivity of Li in the liquid-phase is in the order of 10^–13^ cm^2^ s^–1^, which is two orders of magnitude higher than that in the solid-state. For delithiation, the equilibrium behaviours of CF10–S_pore_ in both TEGDME and EC/DEC electrolytes are identical, *i.e.* the same equilibrium potential and overpotential. This observation is consistent with the solid-state Li–S reaction mechanism since the delithiation process does not rely on Li^+^ ion desolvation so that the type of solvent does not affect the delithiation. A persistent behaviour of the solid-state Li–S electrochemical reaction is the low delithiation capacity in the first cycle (previously shown in Fig. 3 and 5), which is also demonstrated as an inherent property of the sulphur in sub-nano confinements by GITT. Although the exact mechanism is still under investigation, we propose the following explanations: (1) the imposed potential (<3 V *vs.* Li/Li^+^) is insufficient to delithiate Li_2_S within the solid-state environment; (2) degradation of the electrical connection induced by the sulfur volume change.

## Conclusions

In conclusion, we elucidate a mechanism of solid-state Li–S electrochemical reaction in liquid electrolytes enabled by sub-nano confinement of sulphur. Our results demonstrate unambiguous transition of electrochemical behaviours from superficial sulphur to sub-nano confined sulphur, and from sub-nano confined sulphur to sulphur in relatively larger confinements. We clearly demonstrate that the lithiation and delithiation of sulphur in sub-nano confinement is thermodynamically different from conventional liquid phase Li–S reactions. As a result, both ether-based electrolyte and carbonate-based electrolytes are viable for Li–S electrochemical reactions in sub-nano confinement environments since the solid-state mechanism does not involve or require lithium polysulfide dissolution or polysulfide/electrolyte compatibility. Therefore, any Li-ion electrolyte satisfying the electrochemical stability and conductivity requirements should work with the sub-nano confined sulphur cathode. Compared to conventional liquid phase Li–S electrochemical reactions, this solid-state mechanism has the benefit of simplicity, which can provide a new paradigm for future Li–S battery materials design and synthesis. Meanwhile, the large irreversible capacity in the first cycle presents an inherent challenge to the sub-nano confined sulphur, which is currently under investigation in our group.

## Supplementary Material

Supplementary information
