# Supplementary material for: Solid state lithiation–delithiation of sulphur in sub-nano confinement: a new concept for designing lithium–sulphur batteries
Source: Chem Sci. 2015 Nov 10;7(2):1224–32. doi: 10.1039/c5sc03419a (PMC5975833; doi:10.1039/c5sc03419a)
Supplement: Supplementary file 1 [file SC-007-C5SC03419A-s001.pdf]

Electronic Supplementary Material (ESI) for Chemical Science.
This journal is © The Royal Society of Chemistry 2015

Electronic Supplementary Information

Solid State Lithiation-Delithiation of Sulfur in Sub-Nano Confinement: A New Concept for Designing Lithium-Sulfur Batteries

*Chengyin Fu, Bryan M. Wong, Krassimir N. Bozhilov, Juchen Guo**

Experimental

Preparation of Sulfur-Carbon Composites: CF10-S_{60} and CF10-S_{90} were prepared by dissolving a designated amount of sulfur (Sigma-Aldrich) into carbon disulfide (CS_2, Sigma-Aldrich) which were then mixed with porous carbon fibers (Kuraray Chemical Co., Japan). After drying at 60 °C, the mixture was heated at 155 °C at a heating rate of 5 °C min^{-1} in a sealed stainless steel vessel (with glass lining) filled with argon for 10 hours. CF10-S_{pore} was achieved by further heat-treating CF10-S_{60} at 200 °C in flowing argon for 6 hours to completely remove the sulfur deposited on the surface, and CF20-S_{pore}, CF25-S_{pore}, and CF30-S_{pore} were prepared using the same method.

Measurement of solubility of lithium polysulfides: Li_2S (Alfa Aesar) was reacted with a designated amount of sulfur in anhydrous TEGDME (Sigma-Aldrich) or anhydrous EC/DEC (1:1, v/v) (Sigma-Aldrich) to form lithium polysulfide solutions at room temperature (xS + $Li_2S \rightarrow Li_2S_{1+x}$). For example, 0.046 g Li_2S could react with 0.224 g S (1:7 molar ratio) in 10 ml TEGDME by stirring to produce a 0.1 M Li_2S_8 solution with complete dissolution. To measure the solubility of lithium polysulfide in TEGDME, 0.005 M Li_2S_x (x = 4, 5, 6, 7, 8) solutions were first made using the method described above. If the entire solid were dissolved, the concentration would be increased by 0.005 M by adding in required Li_2S and sulfur stoichiometrically. This procedure was repeated with 0.005 M interval until solid precipitation was obtained. To measure the solubility of lithium polysulfide in EC/DEC, 0.001 M of the Li_2S_x solutions were prepared instead of 0.005 M, and the solid was never completely dissolved.

Materials Characterizations: The nitrogen adsorption-desorption isotherms of the carbon fibers were obtained with a Brunauer-Emmett-Teller (BET, Micromeritics ASAP2020). Thermogravimetric analysis (TGA, TA Instruments Q500) was used to measure the sulfur content in the composites. The crystal structure of the sulfur in the composites was characterized with X-ray powder diffraction (XRD, PANalytical Empyrean). The oxidation state of the sulfur was characterized with X-ray photoelectron spectroscopy (XPS, AXIS Ultra DLD). The XPS spectra of lithiated CF10-Spore and CF10-S90 were collected on a Kratos AXIS 165 spectrometer using monochromatic Al Kα radiation (280 W). Transmission electron microscopy (TEM, FEI CM300) coupled with an energy dispersive X-ray (EDX) spectrometer was used to obtain images and elemental mapping of the cross-section of the sulfur-carbon fibers. The fibers were embedded in Spurr resin and let harden overnight at 60 °C. Thin sections with thickness of about 60 nm were then cut normal to the fiber elongation with an RMC ultramicrotome. The individual thin sections were mounted on 3 mm TEM Cu grids covered with lacey carbon support film.

Electrochemical Measurements: The electrodes were comprised of 70 wt% sulfur-carbon composite, 20 wt% carbon black (Super P), and 10 wt% poly(vinylidene fluoride) (Sigma-Aldrich) binder. Aluminum foil (99.45%, Alfa Aesar) was used as the current collector. Two-electrode coin cells with lithium foil (Sigma-Aldrich) as the counter electrode were assembled in an argon-filled glovebox for the electrochemistry analysis. Electrolytes consisting of 1 M lithium bis-(trifluoromethane)sulfonimide (LiTFSI, Sigma-Aldrich) in tetraglyme (TEGDME, Sigma-Aldrich) and 1 M lithium hexafluorophosphate ($LiPF_6$) in EC/DEC (1:1, v/v) (Sigma-Aldrich) were used with a microporous membrane separator (Celgard 2500). The cells were charged and discharged with different cycling currents between 1 V and 3 V (vs. Li^+/Li) using an Arbin battery test station. Cyclic voltammogram (CV) measurements were carried out with a scan rate of 0.1 mV s^{-1} on a Gamry Interface 1000 analyzer. For galvanostatic intermittent titration technique (GITT) experiments, the cells were discharged or charged at 67 mA g^{-1} for 1 hour and then rest for 16 hours. The discharge (or charge)-rest process was repeated until the voltage reached potential winder limits. All the electrochemical measurements were performed at 25°C. The diffusivity of Li was calculated based on the following equation:

$$D = \frac{4}{\pi\tau}\left(\frac{n_m V_m}{S}\right)^2 \left(\frac{\Delta E_s}{\Delta E_t}\right)^2$$

τ is the current pulse time (s); n_m is the number of moles of sulfur in the electrode (mol); V_m is the molar volume of sulfur (cm^3/mol); S is the contact area (cm^2); ΔE_s is the steady-state voltage change due to the current pulse, and ΔE_t is the voltage change during the constant current pulse, eliminating the IR drop.

UV-vis Spectroscopy Experiment: A CF10-S_{pore} electrode and a CF10-S_{90} electrode contained the same amount of sulfur (~10 mg), respectively, were immersed into the TEGDME electrolyte and sealed in two homemade PTFE cylindrical cells with Li counter electrode. The cells were discharged with 80 mA g^{-1} current density to ensure in-depth lithiation. After the lithiation, the TEGDME electrolytes in these two cells were immediately extracted and sealed in the glove-box for UV-Vis spectroscopy analysis with a UV−visible spectrophotometer (Horiba Aqualog).

Density functional theory (DFT) calculations: All DFT calculations utilized second-tier numeric atom-centered basis functions in conjunction with the semi-local PBE functional. In order to account for van der Waals (vdW) effects, the PBE functional was augmented with a vdW correction using a Hirschfeld partitioning of the electron density. Spin-orbit effects were not included in the DFT calculations due to their immense computational effort. All geometry optimizations (optimizing both the cell and ionic positions) were carried out with the vdW-corrected PBE functional until the total electronic energy was converged to 10^{-6} eV and the forces converged to within 10^{-4} eV/Å. Upon convergence to their optimized geometries, all of the material systems were then subjected to additional analyses on the self-consistent electronic density to compute the XPS signals. Specifically, XPS signals were obtained within the initial-state approximation by projecting the density of states onto the basis functions of the individual atoms in the system (according to their atomic angular momenta: i.e. □ = 0 and □ = 1 for s and p orbitals, respectively). Each of the individual atom-projected density of states was then broadened to account for finite temperature effects in order to match the experimental XPS signals.

Discussion on the form of sulfur in the sub-nano confinement: One generally accepted hypothesis is that small sulfur molecules such as S_4 or S_2 exist in the sub-nano pores, which lead to the anomalous electrochemical behaviors. However, this hypothesis may need further studies for the following reason: The percentage of small sulfur molecules should be extremely low in the sulfur under our experimental conditions (near 1 atm pressure and temperature ≤ 200°C): In a review article by Meyer (*Chem. Rev.* 1976, *76*, 367-388), the author estimated that sulfur molecules smaller than S_4 did not exist in liquid sulfur at 155°C (temperature used for sulfur infusion in our study). The overwhelming majority of sulfur molecules at 155°C are still cyclo-S_8 (approximately 80 mol.%) and other long sulfur chains or sulfur rings with atom number higher than 8 due to polymerization. Small sulfur molecules such as S_6 are approximately 1 mol.%, and S_5 is only approximately 0.003 mol.%. In an article by Steudel et al. (Steudel, R.; Steudel, R.; Wong, M. W. *Top. Curr. Chem.* 2003, 230, 117-134), the authors calculated the concentration of sulfur species in the saturated vapor at 500°C based on the experimental thermodynamic data of Rau et al. (*J. Chem. Thermodyn.* 1973, 5, 833): the molar concentration of S_2, S_3, and S_4 are 5.0 mol.%, 1.3 mol.%, and 1.0 mol.%, respectively, and the concentration of S_8 is 33.0 mol.%. Rau et al. in their *J. Chem. Thermodyn* paper also calculated the vapor sulfur species concentration as a function of temperature, which also shows that the concentration of small sulfur molecules is really low in gas phase sulfur below 800K. Based on the description above, we can draw a sound conclusion that the concentration of small sulfur molecules in our experimental condition is extremely low. If we infuse sulfur into the sub-nano carbon pores with extremely low concentration of small sulfur molecules, the only scenarios we could envision that have the majority of pores filled with small sulfur molecules are (1) infusing with extremely large amount of sulfur, which is obviously no true in our case; or (2) generating small sulfur molecules within the pores, of which the only enabling force could be some surface adsorption energy that was high enough to "break off" a few (2 to 4) bonded sulfur atoms from the main molecule and maintaining them as small molecules. In other words, the adsorption energy has to be equivalent to the thermal energy to generate high concentration of small sulfur molecules in vapor phase, which is obviously not the case either. Therefore, we propose that the majority of sulfur in the sub-nano confinement in our study is still cyclo-S_8 molecule. This hypothesis will be investigated in our future work.

Table S1. Surface area and porosity data of the carbon fiber samples.

Sample	BET Surface Area (m^2 g^{-1})	Pore Volume (cm^3 g^{-1})	Average Pore Size (nm)	Pore Size Distribution (nm)
CF10	669	0.359	0.67	0.4 – 1.0
CF20	1075	0.538	0.87	0.4 – 2.0
CF25	1367	0.714	0.99	0.4 - 2.5
CF30	1667	0.863	1.2	0.4 – 3.0

Table S2. Solubility of Li_2S_x (x = 4, 5, 6, 7 and 8) in TEGDEM and EC/DEC (1:1 volume ratio) at room temperature.

	Li_2S_4	Li_2S_5	Li_2S_6	Li_2S_7	Li_2S_8
**TEGDME**	< 0.005 M	< 0.005 M	< 0.005 M	0.04 ± 0.005 M	0.18 ± 0.005 M
**EC/DEC**	< 0.001 M	< 0.001 M	< 0.001 M	< 0.001 M	< 0.001 M

Table S3. XPS peak position and area of sulfur species in the lithiated CF10-S_{pore} and CF10-S_{90}.

Species	CF10-S_{pore}		CF10-S_{90}	
	Position (eV)	Area (%)	Position (eV)	Area (%)
S^{2-}	161.05<br>162.23	67.77	160.89<br>162.41	49.52
S_2^{2-}	162.55<br>163.73	29.62	161.98<br>163.47	27.69
S	164.04<br>165.23	2.61	164.05<br>165.24	22.79

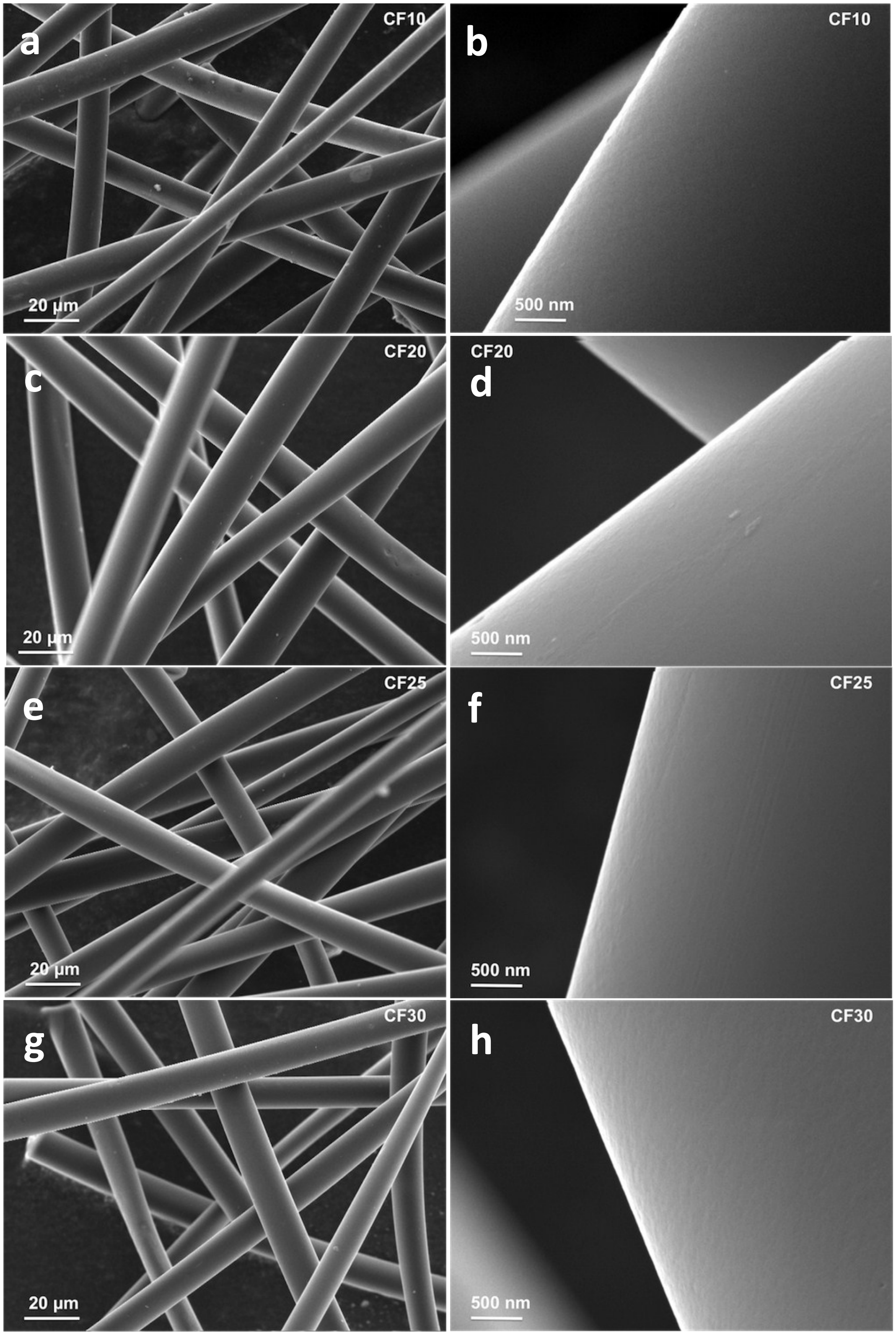

Figure S1. SEM images of (a, b) CF10, (c, d) CF20, (e, f) CF25, and (g, h) CF30 carbon fibers.

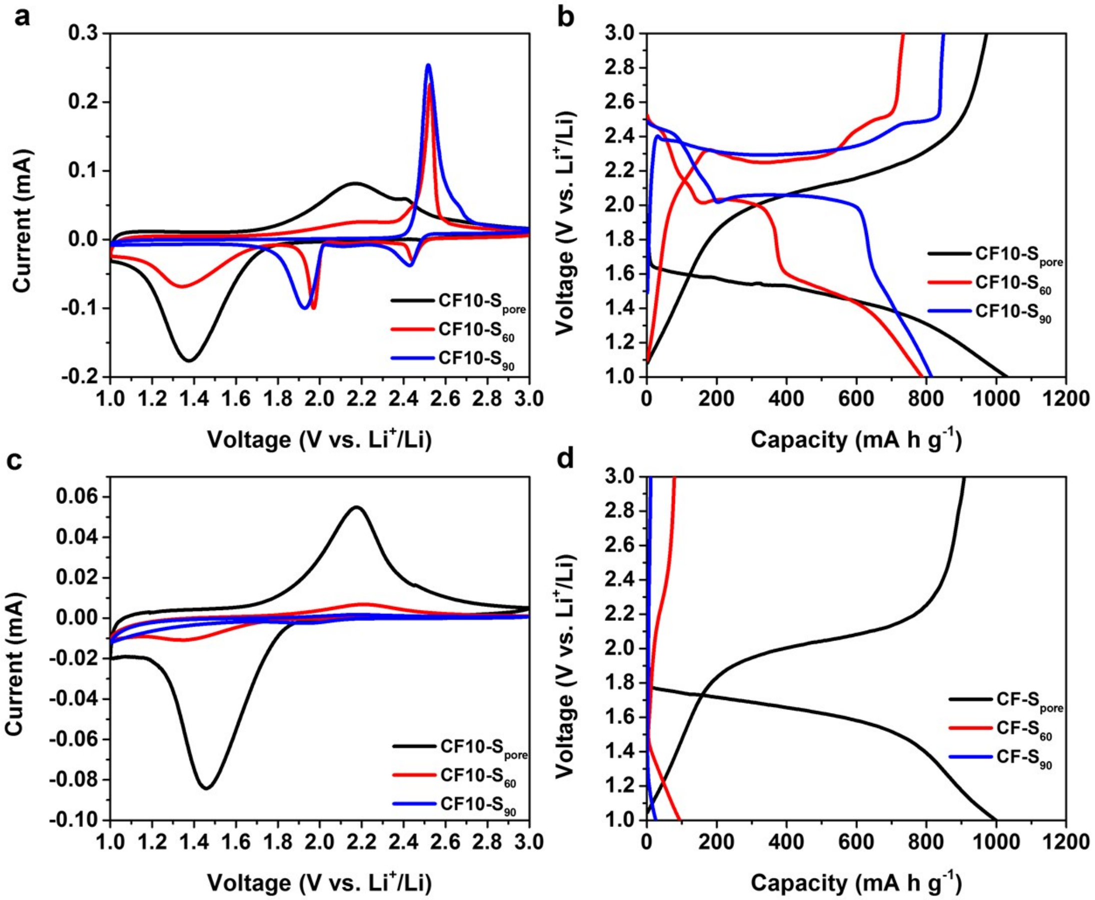

Figure S2. The second CV scans at 0.1 mV S^{-1} and the second GCD curves at 160 mA g^{-1} of CF10-S_{pore}, CF10-S_{60} and CF10-S_{90} in TEGDME electrolyte (a) and (b) respectively, and in EC/DEC electrolyte (c) and (d), respectively.

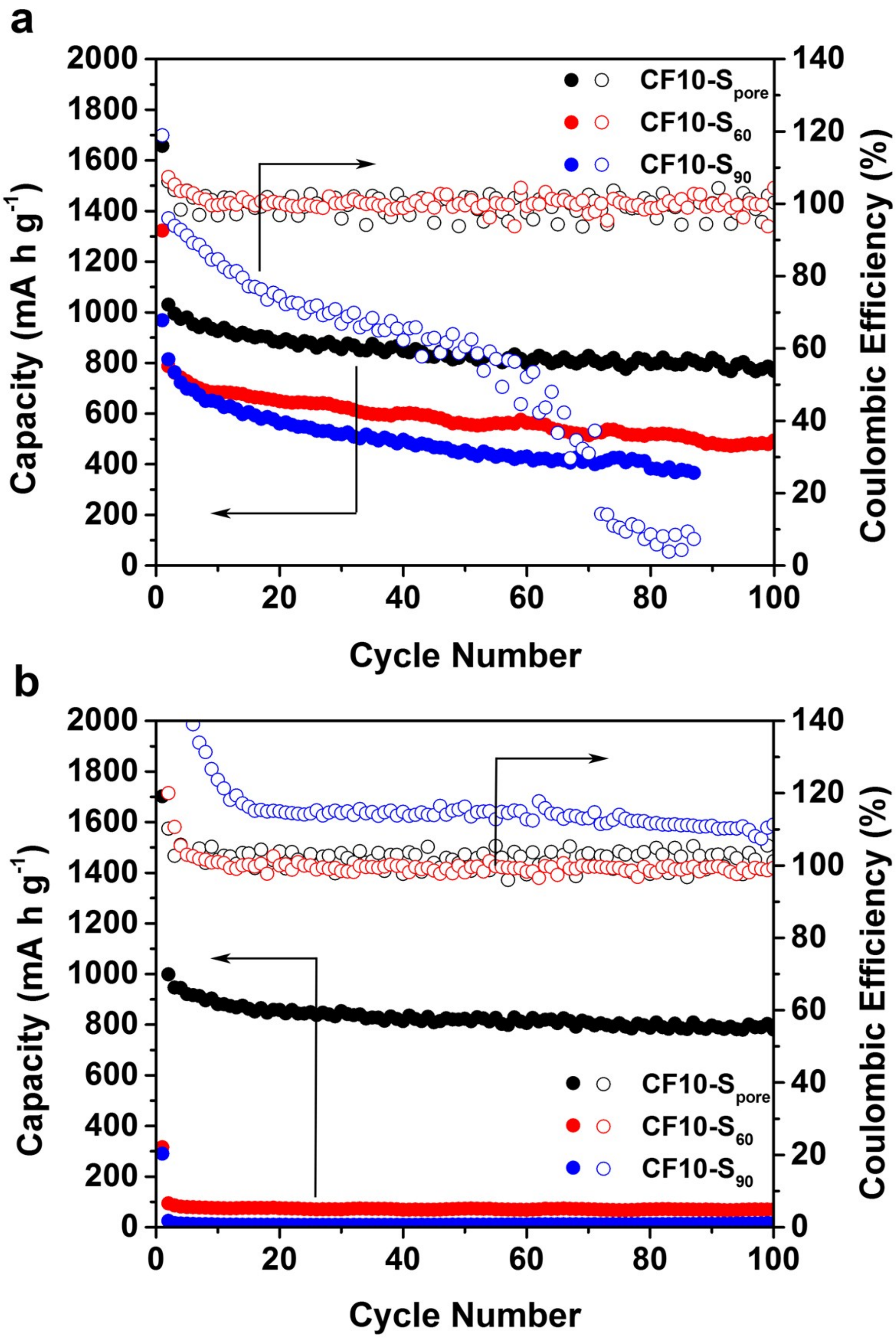

Figure S3. Cycle stability of CF10-S_{pore}, CF10-S_{60}, and CF10-S_{90} at 160 mA g^{-1} in (a) TEGDME electrolyte and (b) EC/DEC electrolyte.

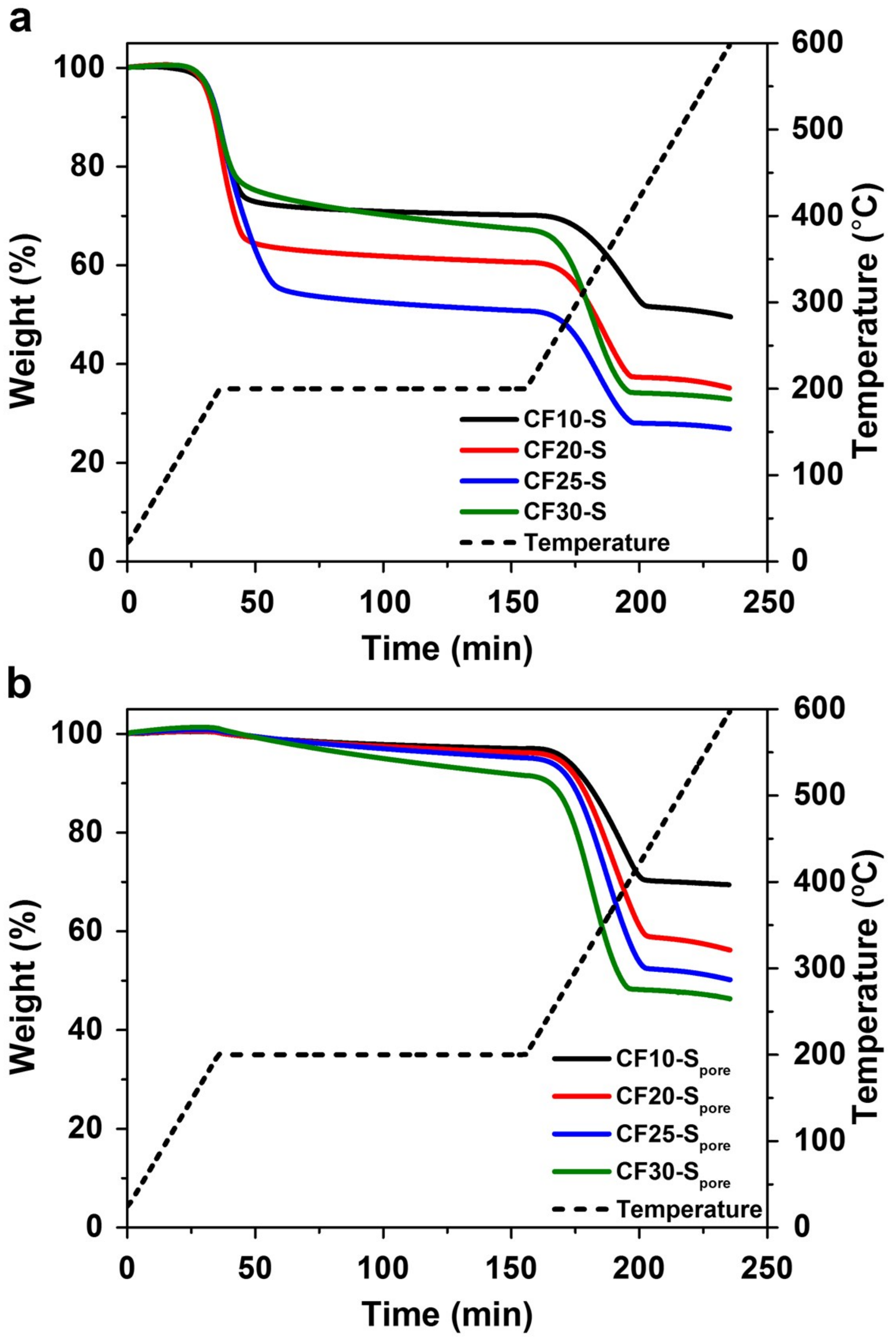

Figure S4. TGA curves of CF10-S_{pore}, CF20-S_{pore}, CF25-S_{pore}, and CF30-S_{pore} (a) before and (b) after superficial sulfur removal via heat treatment at 200 °C.

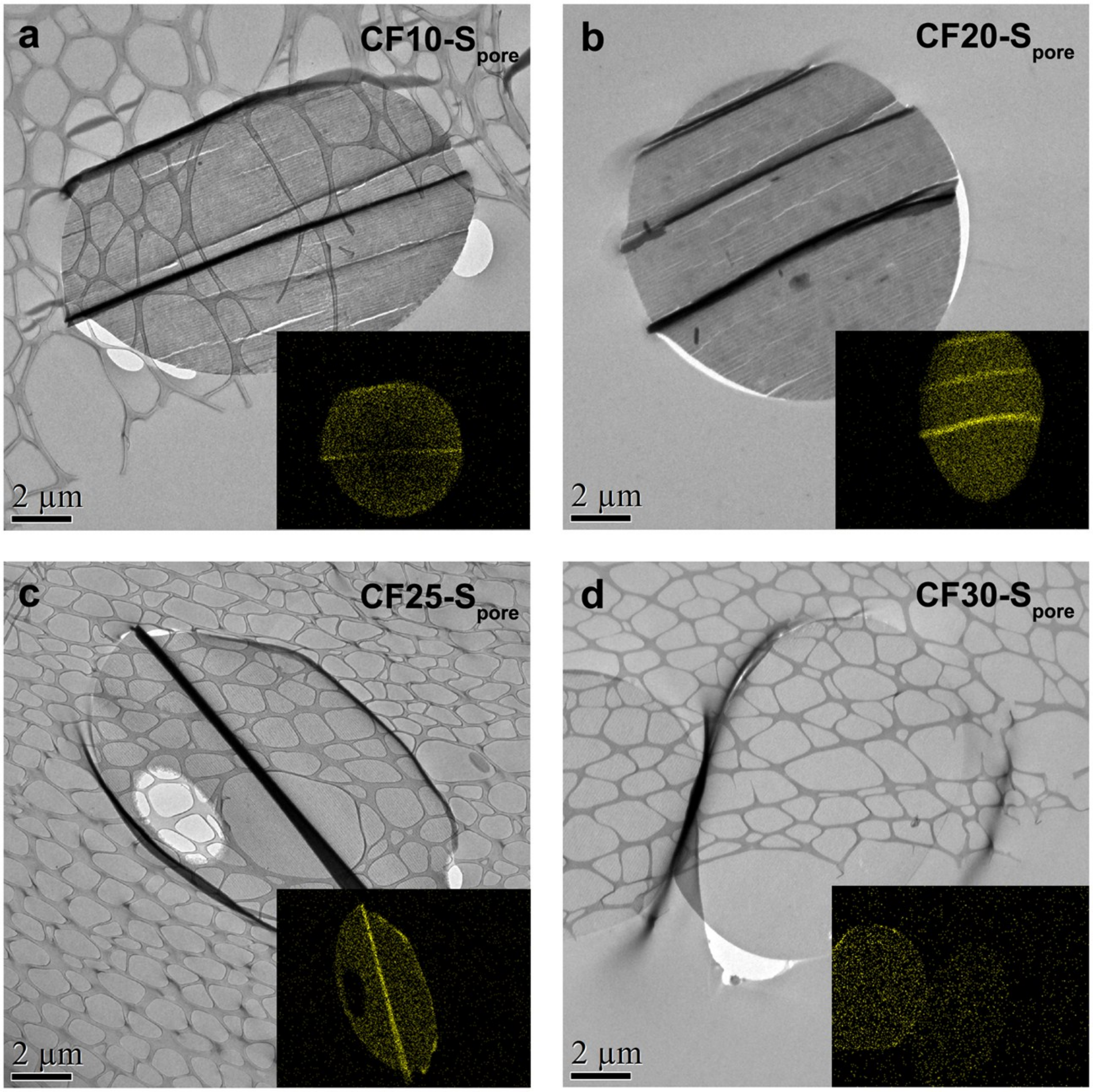

Figure S5. Cross-section TEM image and sulfur elemental mapping (inset) of (a) CF10-S_{pore}, (b) CF20-S_{pore}, (c) CF25-S_{pore}, and (d) CF30-S_{pore}.

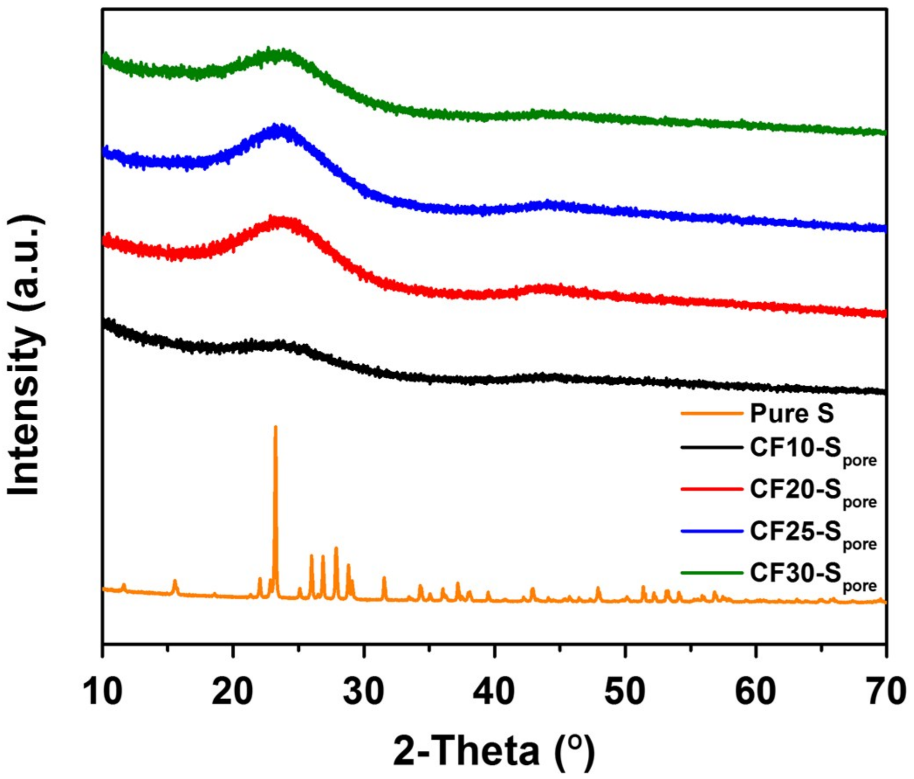

Figure S6. XRD spectra of CF10-S_{pore}, CF20-S_{pore}, CF25-S_{pore}, and CF30-S_{pore}.

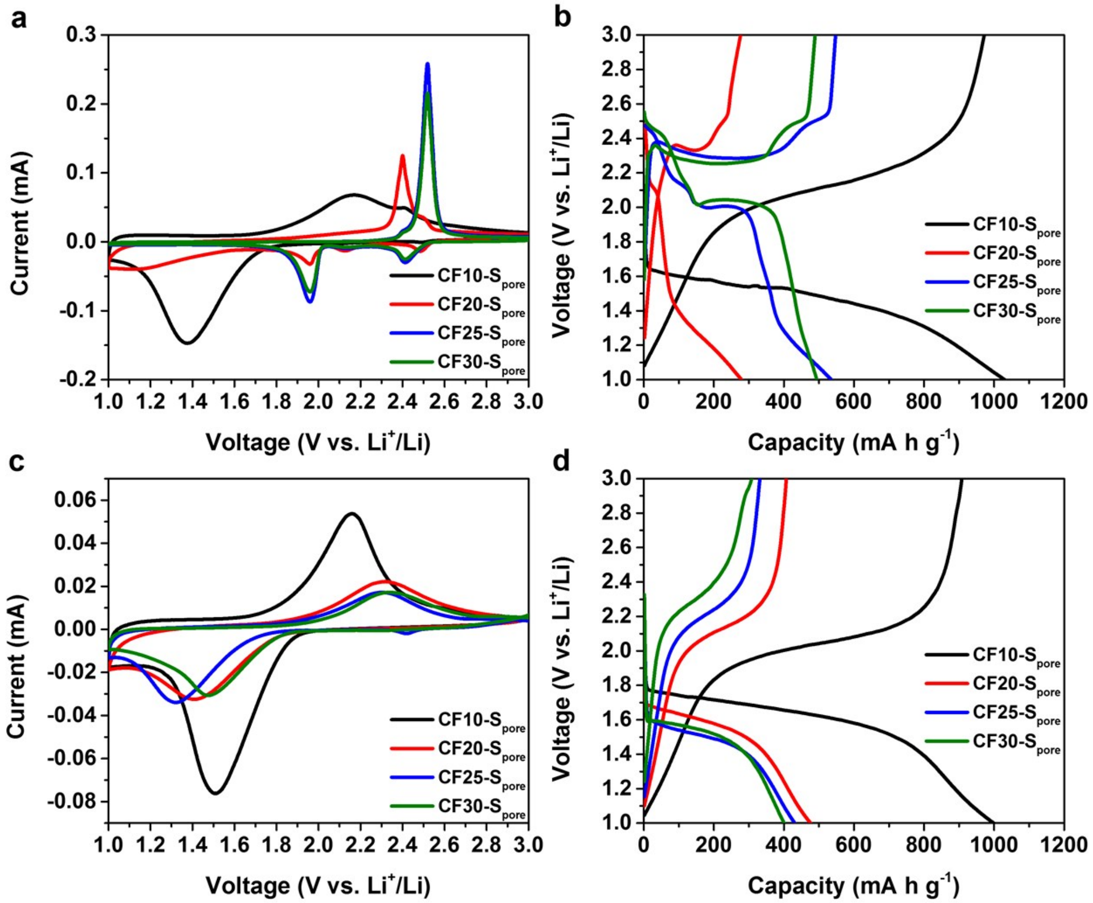

Figure S7. The second CV scans at 0.1 mV S^{-1} and the second GCD curves at 160 mA g^{-1} of CF10-S_{pore}, CF20-S_{pore}, CF25-S_{pore}, and CF30-S_{pore} in TEGDME electrolyte (a) and (b) respectively, and in EC/DEC electrolyte (c) and (d), respectively.

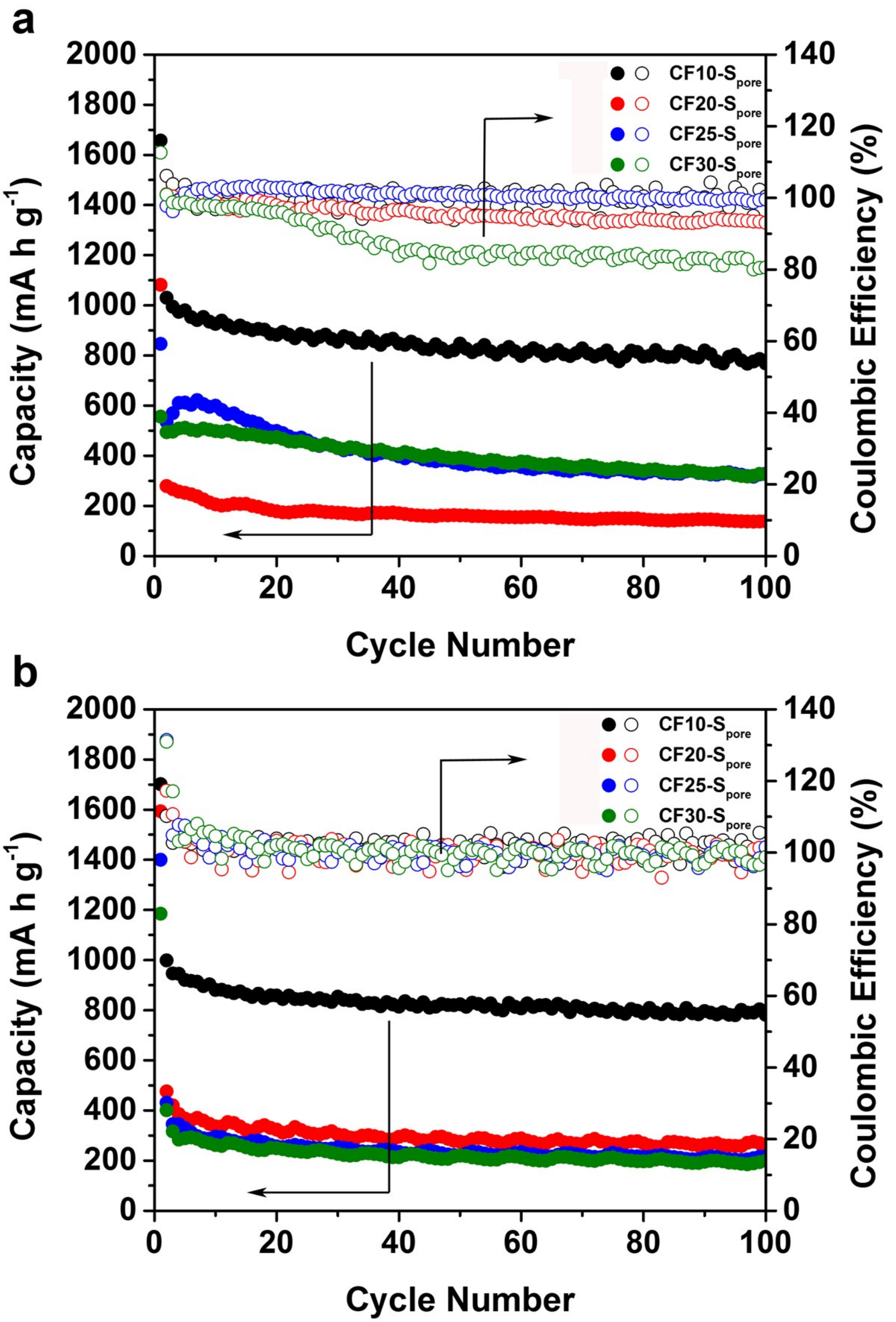

Figure S8. Cycle stability of CF10-S_{pore}, CF20-S_{pore}, CF25-S_{pore}, and CF30-S_{pore} at 160 mA g^{-1} in (a) TEGDME electrolyte and (b) EC/DEC electrolyte.

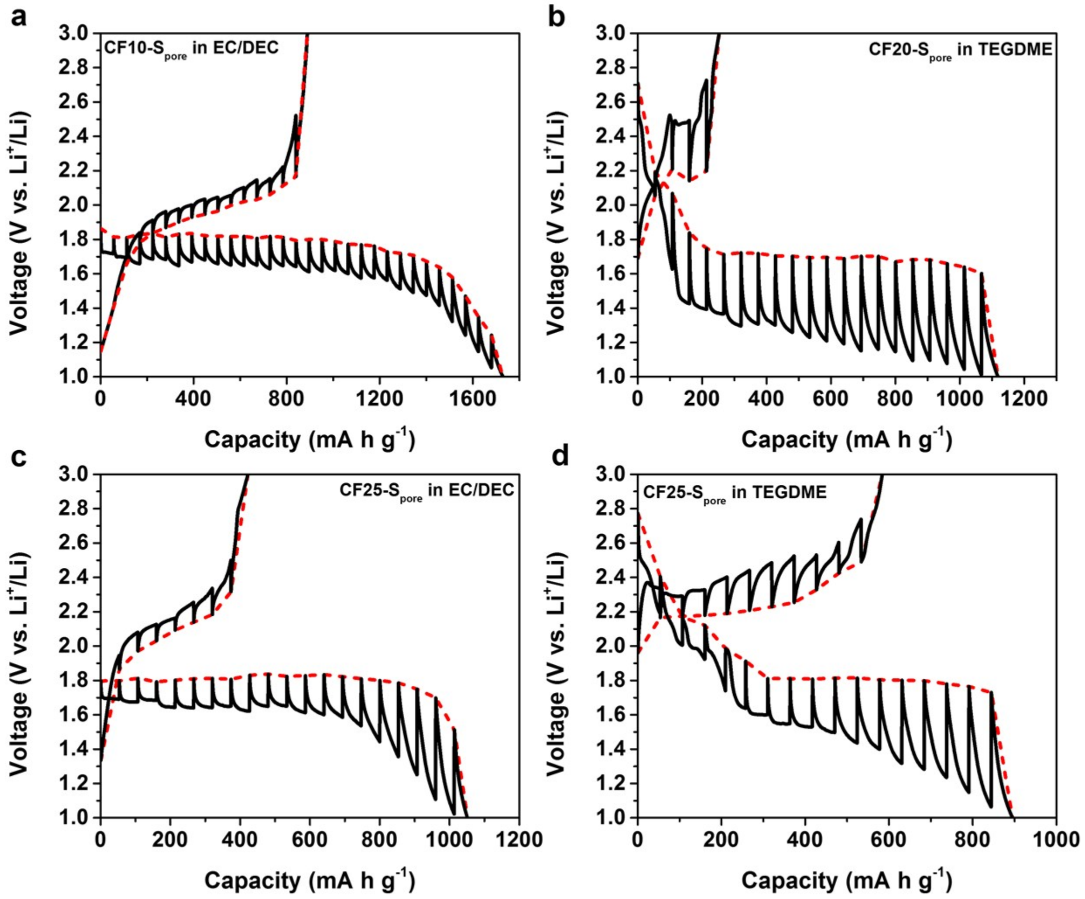

Figure S9. GITT curves of CF20-S$_{pore}$ in (a) EC/DEC and (b) TEGDME electrolytes; and GITT curves of CF25-S$_{pore}$ in (c) EC/DEC and (d) TEGDME electrolytes.

The equilibrium lithiation-delithiation behaviors of CF20-S$_{pore}$ and CF25-S$_{pore}$ in the EC/DEC electrolyte are consistent with that of CF10-S$_{pore}$. However, the capacity decreases and the lithiation overpotential increases simultaneously with increasing sulfur confinement size, most likely due to the kinetic limitation induced by the larger sulfur size. On the contrary, the equilibrium behaviors of CF20-S$_{pore}$ and CF25-S$_{pore}$ in the TEGDME electrolyte are significantly different. The GITT of CF20-S$_{pore}$ in TEGDME initially shows liquid Li-S reaction behavior with short high-potential plateaus followed by a 1.8 V plateau, which indicates a solid-state Li-S reaction. It is also interesting to see that the solid-state lithiation overpotential of CF20-S$_{pore}$ is very high (> 400 mV) leading to a low lithiation working potential. Meanwhile, the delithiation capacity of CF20-S$_{pore}$ is inherently low, and the delithiation curve also shows that only the liquid phase Li-S reaction is reversible. The equilibrium lithiation curve of CF25-S$_{pore}$ in TEGDME shows a more pronounced signature of liquid phase Li-S reactions. The delithiation curve of CF25-S$_{pore}$ in TEGDME also demonstrates liquid phase Li-S behaviors with increased delithiation capacity. However, it needs to be pointed out that the capacity of delithiation in liquid phase Li-S reactions may be artificially high due to polysulfide dissolution.

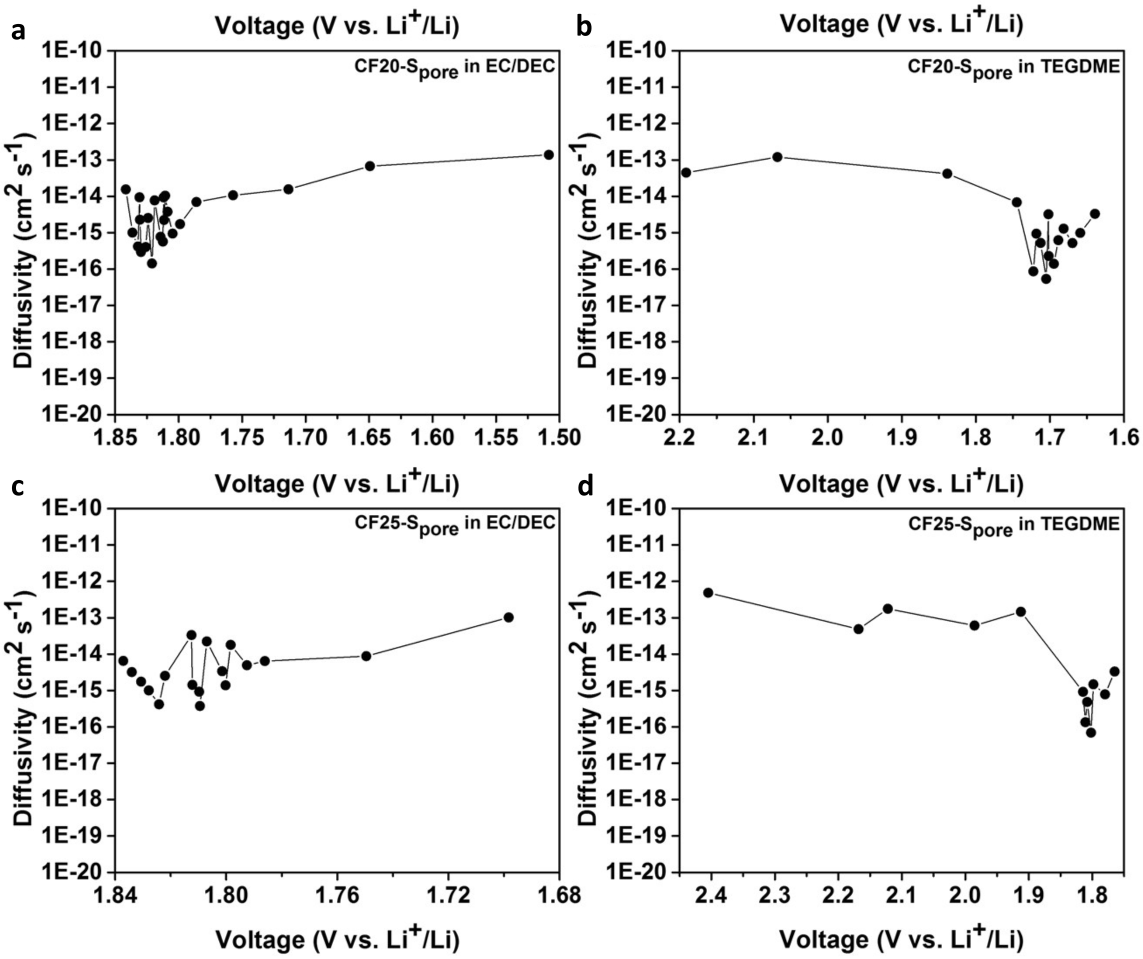

Figure S10. Li diffusivity calculated from the GITT data of CF20-S_{pore} in (a) EC/DEC and (b) TEGDME electrolytes; CF25-S_{pore} in (c) EC/DEC and (d) TEGDME electrolytes.
